# Classical Biological Control of Invasive Legacy Crop Pests: New Technologies Offer Opportunities to Revisit Old Pest Problems in Perennial Tree Crops

**DOI:** 10.3390/insects6010013

**Published:** 2014-12-23

**Authors:** Mark S. Hoddle, Keith Warner, John Steggall, Karen M. Jetter

**Affiliations:** 1 Department of Entomology, University of California, Riverside, CA 92521, USA; 2 Center for Science, Technology, and Society, Santa Clara University, CA 95053, USA; E-Mail: kwarner@scu.edu; 3 California Department of Food and Agriculture, Sacramento, CA 95814, USA; E-Mail: jsteggall@cdfa.ca.gov; 4 UC Agricultural Issues Center, University of California, Davis, CA 95616, USA; E-Mail: jetter@primal.ucdavis.edu

**Keywords:** climate matching, ecological niche modeling, invasive species, molecular analyses, natural enemies

## Abstract

Advances in scientific disciplines that support classical biological control have provided “new tools” that could have important applications for biocontrol programs for some long-established invasive arthropod pests. We suggest that these previously unavailable tools should be used in biological control programs targeting “legacy pests”, even if they have been targets of previously unsuccessful biocontrol projects. Examples of “new tools” include molecular analyses to verify species identities and likely geographic area of origin, climate matching and ecological niche modeling, preservation of natural enemy genetic diversity in quarantine, the use of theory from invasion biology to maximize establishment likelihoods for natural enemies, and improved understanding of the interactions between natural enemy and target pest microbiomes. This review suggests that opportunities exist for revisiting old pest problems and funding research programs using “new tools” for developing biological control programs for “legacy pests” could provide permanent suppression of some seemingly intractable pest problems. As a case study, we use citricola scale, *Coccus pseudomagnoliarum*, an invasive legacy pest of California citrus, to demonstrate the potential of new tools to support a new classical biological control program targeting this insect.

## 1. Introduction

Invasive species pose major ecological and economic threats to agriculture worldwide [1]. Classical biological control (biocontrol), the introduction of co-evolved natural enemies to control invasive pests [2], is an alternative approach to using pesticides for suppressing pest populations to less damaging levels. Several steps are involved in developing a biocontrol program targeting an exotic pest and these typically involve: (1) identifying the general area of origin of the pest, and if possible, pin-pointing a specific source region within this much larger range; (2) conducting foreign exploration for natural enemies, that is, executing surveys within the home range of the pest for natural enemies that attack the target and returning these biocontrol agents under permit to an approved quarantine facility; (3) Conducting host range (*i.e.*, determination of the number of species likely to be attacked by the natural enemy) and host specificity (*i.e.*, natural enemy preference for different species) tests in quarantine to determine the risk to non-target species that potential natural enemies may be exposed to, should they be released from quarantine; (4) producing natural enemies in mass, releasing and establishing them. In this phase, natural enemies that are cleared for release from quarantine by regulatory authorities are mass reared and released into areas where the target pest is present. The intent is to establish permanent self-sustaining natural enemy populations that will disperse and track the pest’s distribution; (5) Monitoring the spread and impact of the natural enemy on the target’s population densities.

In response to concerns raised about procedures for locating and selecting safe and efficacious natural enemies for biological control programs [3], biocontrol practitioners have adopted and developed new selection and evaluation tools that have the potential to improve the precision, efficacy, predictability, and safety of biocontrol programs [4,5]. These developments are particularly important when the success rate of a biocontrol project—that is complete suppression of a target pest with introduced natural enemies—is considered, as it is low, averaging around 10%–12% [6].

These “new” tools have the potential to not only improve successful biocontrol of new invasive pests, but they can be applied retroactively to “legacy” pests, which are exotic pests that have been present in cropping systems for a significant amount of time. We suggest from experience that exotic pests that have been present and problematic in crops for >25 years can be considered legacy pests and those establishing prior to 1990 may be well suited for investigation with new tools. Work on biocontrol of legacy pests tends to diminish over time as crops acquire new pest species and attention is focused on mitigating new problems and re-engineering existing integrated pest management (IPM) programs to accommodate new pests. With the passage of sufficient time, legacy pests may eventually be considered “native”, because pest managers have become accustomed to their presence. Some legacy pests may have been targets of past classical biocontrol programs that were unsuccessful (see Section 4.1 for a case study on citricola scale, *Coccus pseudomagnoliarum* [Kuwana] [Hemiptera: Coccidae]). However, developing new biocontrol programs for legacy pests using new technologies that may not have been available at the time of invasion may now be feasible and could significantly reduce perceived risk when revisiting these past targets. Further, successful biocontrol programs for legacy pests may help reduce agricultural pesticide use, especially in California, an agriculturally intensive state that is dependent upon pesticides for crop protection. In 2012 alone, 84,368,181 kg of pesticides were applied in California [7]. It is acknowledged that major improvements in pesticide use are still needed, especially the identification and development of alternative control approaches [8], of which biocontrol is an obvious candidate for increased use for invasive pest management. Here, we suggest that biocontrol of certain legacy pests, including pests that were targets of failed biocontrol programs, deserve to be revisited because advances in theory and techniques may make these legacy pests suitable targets for classical biocontrol. In support of this proposal, we review some of these new tools and their applicability to classical biocontrol. Our application focus for these new tools is on legacy pests of perennial crops in California as these species may have invaded prior to the availability of some of these new technologies, and long-lived tree crops provide stable habitats where biocontrol programs may be more likely to succeed. The following sections are divided into two categories; (1) Pre-introduction research which encompasses efforts to locate natural enemies from the pest’s home range, maintaining genetic diversity of collected natural enemies, and assessing their host range; and (2) post-introduction research addressing natural enemy release strategies, resource availability, and mutualisms that are antagonistic to natural enemies. 

## 2. Pre-Introduction Research on Biological Control Agents

### 2.1. Where to Search for Natural Enemies for Use in Classical Biological Control? Molecular Tools for Identifying Pest and Natural Enemy Species

DNA-based tools are now indispensable technologies in the development of modern classical biological control programs. Molecular approaches are being used with increasing frequency to unambiguously identify pest [9,10], natural enemy species [11] and biotypes [12], and to better understand relationships amongst species (*i.e.*, systematics). The accurate taxonomic identification of pest and natural enemy species has long been recognized as an essential first step in developing successful biological control programs [13,14]. Failure to correctly identify the pest, its natural enemies, or both can cause project failure or significantly stall forward momentum [14,15]. Correct taxonomic identification and an understanding of relationships between pertinent species is important to biological control because this information will guide foreign exploration efforts based on an understanding of evolutionary areas of origin and biogeography [16]. Correct pest and natural enemy identification assists with understanding the evolution of host specificities and investigations into life history studies, which will directly influence mass production efforts of the pest and its natural enemies [13]. Taxonomy and systematics have provided the concept for the existence of cryptic species, organisms that may differ in biology, ecology, and behavior, but cannot be reliably identified using physical traits. However, variation at the molecular level may be detectable and could be sufficiently great enough to warrant recognition as separate biological entities even when populations are sympatric [9].

In the invaded range, species identification can be complicated when closely related pest species are brought into sympatry and inter-breeding results in unique hybrids that may not exist in the native range [16]. Detecting this possibility can be crucial as natural enemies that have co-evolved with particular species may be unable to recognize and attack hybrid pest species in the invaded range [16,17]. Accurate species identifications may be further complicated by a paucity of experts or lack of interest by taxonomists that specialize in key groups that are the focus of a research program [18,19].

DNA analyses, in particular DNA “barcoding” can be very useful in overcoming this taxonomic impediment to species identifications as it allows “non-experts” to separate and identify putative species based on DNA-level differences. DNA barcoding assumes that species are characterized by unique genetic sequences and that molecular differences at the DNA-level are greater between species than within species because sequence divergences are ordinarily much lower among individuals of a species than between closely related species [20,21]. Genetic differences can be used to identify species, unnamed species may be referred to as operational taxonomic units because DNA sequences, not names, are used to define species, and these DNA data can be used to construct phylogenetic trees if needed. This molecular approach may aid the discovery of cryptic species, biotypes, and new species, and has the potential to resolve conflicts over synonymies that are intractable with traditional morphological approaches [20,22].

The use of morphology and molecular analyses for species identification can be complementary [23] and has proven very useful in pest species identification [9,24]. Despite these potential benefits for identifying and separating unnamed species, DNA barcoding for this purpose is not universally accepted. Opponents state that barcoding is not a robust alternative to conventional taxonomic techniques because barcodes cannot provide a physical description of type specimens, an essential requisite needed for naming, describing, and identifying new species [25,26]. For biological control programs, a combination of identification tools should be used to identify novel pest and natural enemy species. For new pest and natural enemy species, descriptions and names published in peer reviewed journals will likely be required before regulatory agencies grant permission to release natural enemies from quarantine for use in classical biological control programs.

### 2.2. Where to Search? Climate Matching Tools

Selecting areas for foreign exploration for natural enemies needs to take into consideration similarities between the climate of the source area for the biocontrol agents and the receiving area for their release. The underlying logic is that natural enemies should be pre-adapted to the climatic conditions in which they will be expected to function [27]. Climate may correlate with potential spread of natural enemies in new areas as they track target populations [28,29], and regional climatic differences can affect natural enemy efficacy and their ultimate distribution, which may or may not completely cover the pest’s distribution in the invaded range [30]. Stiling’s [31] meta-analyses on natural enemy failures estimated that approximately 35% of biocontrol introductions or programs may have been unsuccessful because of climate related factors. Climatic mismatch, especially extremes of temperature, rainfall, humidity, and photoperiod, is presumed to be an important factor limiting establishment, spread, and impact of natural enemies, and has likely been a cause of failure in some biological control programs [27,30,31,32,33,34].

Species distribution models (e.g., CLIMEX, bioSIM, BIOCLIM, DOMAIN, HABITAT, and MAXEnt [35,36]) driven by bioclimatic data can be used to generate pest distribution maps [36,37] that can help focus natural enemy searches on areas of the pest’s native range that are climatically similar to the intended receiving range for natural enemies [38,39,40,41,42,43]. Laboratory experiments can be useful alternatives to computer simulations for assessing the possible effects of temperature when evaluating the biocontrol potential of natural enemies. For example, Wang *et al.* [44] compared the thermal performance of the olive fruit fly, *Bactrocera oleae* (Rossi) (Diptera: Tephritidae), and its co-adapted parasitoids to identify natural enemies most suited to the climate in areas of California where olives are grown. Geographic information system (GIS) models using natural enemy degree-day data have been used to predict geographic overlap between an invasive pest and natural enemies used in biological control programs and model predictions fitted well with known geographic distributions for key biocontrol agents [45,46].

Studies consistently demonstrating the importance of climate matching for guiding foreign exploration efforts and subsequent natural enemy selection are not plentiful, and the importance of this approach appears to be quite limited in some situations [47]. However, retrospective climate matching analyses for aphelinid parasitoids released in the United States for biological control of the silverleaf whitefly, *Bemisia argentifolli* Bellows and Perring (Hemiptera: Aleyrodidae), demonstrated the importance of climate matching as an indicator for predicting species establishment [48]. Goolsby *et al.* [48] demonstrated that the average climatic match index value for parasitoids establishing in a new area was ~75%, while for parasitoids failing to establish, the average climatic match index was ≤67% [48]. However, high climatic match indices did not guarantee establishment, and some parasitoid species with climatic match indices of 80% failed to establish indicating that factors other than climate could be important in affecting establishment even though suitable agents with good climatic tolerance were identified [48]. Some mechanisms that might prevent establishment of climatically well-matched natural enemies may include attack by generalist predators, lack of genetic diversity, insufficient numbers released, or impoverished habitat [49].

### 2.3. How Many and Which Natural Enemy Species to Release? Modeling Tools

The number of natural enemy species to release against a target pest to maximize the likelihood of successful control is a controversial issue [50]. Meta-analyses suggest that significant pest suppression is typically achieved by a single natural enemy species [50,51] and that cumulative control effects are often not achieved by establishing multiple agents [51]. Instead, releasing multiple agents with the intent of the “best” one establishing and providing control has been termed the “lottery” approach and may have unintended detrimental outcomes should a sub-optimal natural enemy species establish itself [51]. For example, with competing natural enemy species, antagonism may result in effects on the target being less than additive, and this deleterious outcome is most apparent when the same resource (e.g., life stage) is attacked [52]. Thus, special care needs to be taken if releases of additional natural enemy species are planned [53]. Knowing which natural enemy to select for host range testing (see below) and eventual release from multiple agents discovered during foreign exploration is difficult and may require laboratory and field studies in either the home range (assuming there is no available published literature on the complex of interest) or quarantine (a daunting challenge due to regulatory and space constraints).

Population and life history models of target pests may reveal susceptible life stages, thereby guiding natural enemy selection [54,55]. One characteristic that appears to be beneficial is host feeding (*i.e.*, the female parasitoid consumes host hemolymph thereby killing it), especially if the life stage that is fed upon is different to that used for oviposition and if host feeding rates are high [56]. Modeling of host feeding and parasitoid reproductive mode (*i.e.*, synovigeny) indicates that consuming hosts does not destabilize parasitoid-host population dynamics [57], a result that supports empirical observations [56]. Host feeding may therefore be regarded as a desirable natural enemy trait, one that is particularly relevant for parasitoids attacking homopteran pests [56]. Models may help identify situations when negative effects could occur that may not be anticipated, such as when two different parasitoid species attack different life stages of the same pest [58]. In some instances the superior competitive natural enemy species may not provide the lowest overall pest densities [58].

Life history modeling can also inform post-release management strategies [59], with results being used to predict the impact of natural enemies on target populations [4,60]. Model types investigating population regulation by natural enemies vary significantly [61,62], and one family, matrix models, can be developed to incorporate effects of pest density and combinations of environmental variables [63]. These models have demonstrated that natural enemy activity sometimes causes significant declines in pest densities [63]. However, as the number of variables increases, especially with respect to natural enemy species interacting with the pest, resulting population dynamics are complicated owing to non-linear effects (e.g., natural enemy behaviors) and model outcomes may be sensitive to initial starting population densities [64].

### 2.4. Tools to Preserve Genetic Variation in Natural Enemies Collected from Foreign Exploration

All classical biocontrol programs import natural enemies for release into a new range where they have not existed previously. During this process, material collected from foreign exploration is returned to quarantine where it is reared to undergo host range testing and to remove potential hyperparasitoids or microbial pathogens that attack imported natural enemies. During the importation-rearing process in quarantine, genetic variation in collected material may be lost due to genetic drift, inbreeding, and accidental selection for traits that favor laboratory rearing. Collectively, these genetic changes may result in poor field performance after release because natural enemies fail to adapt adequately to their new environment [65,66,67].

To preserve genetic variation in laboratory colonies, it has been proposed that the establishment of single family lines (referred to as isofemale lines where the male and female offspring of a single mated female are mated and the population in the cage is the product of these repeated sib-matings) can minimize genetic loss thereby preserving a larger amount of genetic variation that is captured during initial field collections [66]. This preservation may occur because inbreeding causes alleles to become fixed in isofemale lines. These alleles are preserved as long as the lines are maintained and this “fixing” process counteracts the effects of laboratory selection, as there is no genetic variation for natural selection to act upon [66]. Small populations consisting of several founders that are repeatedly inbred show genetic deterioration when maintained for multiple generations in the laboratory [67]. However, fitness can be increased when multiple inbred populations are “pooled” and hybrids are produced from random matings between individuals of these formerly isolated inbred lines [67].

Maintenance of isofamilies in quarantine as a means to preserve genetic material was employed for *Tamarixia radiata* (Waterston) (Hymenoptera: Eulophidae) a nymphal parasitoid of Asian citrus psyllid, *Diaphorina citri* Kuwayama (Hemiptera: Liviidae),that was collected from Punjab Pakistan. Seventeen isofamily colonies representing different collection localities and dates were maintained in quarantine and the use of mating cages to cross material from these isofamily lines was employed to generate “hybrid” offspring for field releases with the objective of reconstituting genetic variability in released individuals [68]. Additionally, genetic markers for assessing and maintaining genetic variation for natural enemies in quarantine can be used to examine the effects that multi-generational rearing has on genetic diversity or for assessing the efficacy of quality control practices [69]. Together, these two approaches—isolines and molecular-level diagnostic assessments—may help to preserve natural enemy vigor in quarantine, thereby minimizing genetic bottleneck effects.

### 2.5. Tools for Evaluating the Host Range and Host Specificity of Natural Enemies

A recent and significant development in arthropod biological control has been the development and utilization of host range tests for entomophagous natural enemies (*i.e.*, predators and parasitoids). The motivation for these pre-release quarantine tests has come from field evidence indicating that some entomophagous natural enemies have had significant and unintended impacts on some non-target species, often native insects [70,71]. These types of safety tests have been routinely employed to determine host specificity for weed biological control agents, and the fundamentals underlying tests from weed biological control have been used to develop new theory and test designs for evaluating the host range and specificity of arthropod natural enemies [72]. Briefly, host specificity tests are designed to determine the safety of a natural enemy by evaluating its preference or specificity when presented with a variety of non-target species. Its subsequent ability to develop on these non-target species is evaluated and compared to attack rates, development success, and adult fitness when reared on the target species. Specificity is a measure of preference or intensity of attack on a species while host range indicates the number of species that are vulnerable to attack, which is a metric of safety. For example, a parasitoid may have a high preference for one or two species (e.g., the target and a congeneric species) but is unable to attack other non-targets. This result indicates high host specificity and narrow host range, very desirable characteristics of a biological control agent as it indicates high host fidelity, and this may translate to high impacts on the target species and low risk to non-target species. There are two types of tests that are run to determine the specificity and hence the environmental safety of a natural enemy: (1) choice tests and (2) no choice tests. Choice tests provide the natural enemy with a “choice” between the target pest and non-target species, and the attack rates and subsequent emergence of adult parasitoids are determined following a standardized exposure period. Results indicate which species are most preferred for attack.

No choice tests present just one species for the parasitoid to attack. Results indicate what is likely to happen under extreme conditions when alternative hosts, especially the target, are absent and represent conditions that a natural enemy may encounter when it finds itself in habitat with no target prey. When taken together, the number of target and non-target hosts attacked under choice and no choice conditions provide data on attack rates on different species and these data can be used to assess the level of risk that a candidate natural enemy poses to the environment [38].

A prevailing view in arthropod biological control was, until recently, that such tests were unnecessary as non-targets impacts, if they occurred were inconsequential, and a broad host range for entomophagous natural enemies was a desirable attribute. The theory underpinning this idea was that generalist natural enemies are more likely to establish and polyphagy is beneficial as it maintains natural enemy numbers at high densities when pest populations are low. This idea accepts non-target impacts as being necessary, as natural enemy populations need to be maintained in a constant elevated state of preparation to exert rapid control of pest populations should they rebound.

This concept has been challenged, and the argument for greater use for host-specific natural arthropod enemies has been brought up not only from an environmental safety perspective [73,74] but also because host specific natural enemies, especially parasitoids, are more likely to establish and provide some level of control [75,76]. The most successful natural enemies tend to be those with high host specificity and narrow host ranges, as they are focused almost solely on locating and killing the target pest, and they pose minimal threat to non-target species [73,77]. Meta-analyses also indicate that host specificity may exert a stronger establishment effect than propagule pressure, which is usually considered a major determinant of establishment success [76].

## 3. Post-Release Barriers Affecting Natural Enemy Establishment

### 3.1. Propagule Pressure and Establishment Likelihood

A significant determinant underlying successful natural enemy introductions that have gone through genetic bottlenecking processes is the number of individuals released. This is referred to as propagule pressure, and it is a combination of the number of individuals released (*i.e.*, propagule size) and the number of release events (*i.e.*, propagule frequency). Increased propagule pressure (*i.e.*, the combination of propagule size and frequency) enhances the probability of establishment because adverse demographic (e.g., Allee effects) and genetic effects (e.g., inbreeding) are lessened when large numbers are released [78].

If steps are not taken to mitigate deleterious genetic effects that can occur in laboratory colonies, these problems may be amplified when biocontrol agents are released into new areas. This may occur because a fraction of individuals released likely reproduce which decreases the size of the founder population further, and the genetic variation associated with smaller than expected founder populations declines as a consequence [79].

Modeling of natural enemy release strategies has generated “rules” of thumb which consistently demonstrate that either a few “large” or many “small” releases are best [80,81], and release thresholds (*i.e.*, minimum number of individuals per release) may depend on natural enemy biology, the number of available agents for release, and stochastic environmental effects (e.g., temperature, rain, or host phenology) [82,83]. Field studies have verified the importance of propagule pressure on natural enemy establishment likelihood [84,85], and this is a direct measure of a program’s persistence in attempting to establish biological control agents. For example, large numbers of natural enemies released (*i.e.*, >30,000) resulted in natural enemy establishment approximately 80% of the time, releases of less than 5000 individuals had colonization rates of 10%, and intermediate release numbers had a 40% establishment rate [80]. Release frequencies of more than 20 attempts of more than 800 individuals were more likely to establish, and establishment likelihood appeared to be low when <10 releases were made of fewer than 800 individuals [80]. To increase the likelihood of establishment, these generalizations suggest that significant effort should be invested in making multiple releases of relatively large numbers of natural enemies.

### 3.2. Providing Resources to Natural Enemies

An emerging area that is receiving increasing attention is conservation biological control, the deliberate act of providing shelter and food resources (e.g., flowering plants, which provide nectar) with the goal of attracting, retaining, and promoting the efficacy of natural enemies in cropping systems [86,87]. Laboratory studies have documented the benefits of plant-provided resources, which may be essential for natural enemy survival [88]. These laboratory-demonstrated benefits have been transferred to agricultural systems where increased plant diversity can improve pest control because natural enemies have access to nutritional resources that have been deliberately provided to increase their efficacy [89,90].

Failure to provide food resources pre-release or post-release, may have contributed to natural enemy establishment failure. Scarcity of resources (e.g., floral resources, hosts, and shelter), especially in the initial introductory phases of natural enemy releases when stochastic (e.g., adverse weather) or Allee effects (e.g., mate limitation) can adversely affect the persistence of small highly localized incipient populations during establishment phases. Consideration of resource provisionment may warrant incorporation into biocontrol projects targeting legacy pests. For example, the codling moth, *Cydia pomonella* (L.) (Lepidoptera: Tortricidae), is a legacy pest in California that attacks the fruit of apples, pears, and walnuts. This important pest established in the western USA around 1872 and is the subject of renewed interest for classical biocontrol [91]. *Mastrus ridibundus* (Gravenhorst) (Hymenoptera: Ichneumonidae) is a biocontrol agent of the codling moth, and choice of pre-release diet had a significant effect on this parasitoid’s field dispersal [92]. Providing carbohydrate (e.g., honey-water) and protein sources (e.g., Nu-Lure), or access to target prey for host feeding in the laboratory prior to release of natural enemies has been demonstrated to increase longevity and reproductive output and improve host finding for several different natural enemy species [89,93,94].

### 3.3. Disruptive Mutualisms and Biotic Resistance

Another impediment to successful biological control can be invader-invader interactions that disadvantage the natural enemy. In this instance, reciprocal positive interactions between invasive species amplify invader-specific impacts. One of the best known of these invader mutualisms in biological control are those that ants develop with invasive honeydew-producing pest insects like aphids, scales, whiteflies, mealybugs, and psyllids. Associations between ants and honeydew producers result in a positive population-level feedback system where ant colonies benefit from harvesting nutrient-rich honeydew, the sugary waste product produced by phloem feeding pests, and honeydew producers benefit from protection by patrolling ants, which reduces mortality rates from natural enemy attacks [77,95,96,97,98]. Non-honey dew producing insects can also benefit from increased ant activity that results from tending honeydew producers, as natural enemy activity directed at these non-honeydew producing pests declines as well because of ant presence [96].

Biotic resistance is the degree to which resident species in the targeted ecosystem either inhibit establishment or reduce natural enemy efficacy [99]. For example, resident predator guilds may engage in intraguild predation. This is the killing and eating of competitors (*i.e.*, predatory or parasitic natural enemies) that share a common prey item (*i.e.*, the pest) of which one prey species is the introduced natural enemy intended for the biological control of a target pest [99,100,101,102,103]. Some estimates suggest that biotic resistance can be significant and may have adversely affected outcomes for up to 20% of classical biological control programs [31].

Assessing the effects of invader-invader mutualisms or biotic resistance in advance of releasing natural enemies from quarantine is difficult. Some negative effects can be anticipated based on long historical experience and preemptive measures can be taken. This approach is being recommended for the classical biological control of Asian citrus psyllid, *D. citri*, to protect the nymphal parasitoid *T. radiata* from invasive honeydew collecting Argentine ants, *Linepithema humile* (Mayr) (Hymenoptera: Formicidae), in urban citrus in southern California USA. With increasing urban-agriculture interfaces, backyard trees in urban interface areas function as refuges for natural enemies rather than acting solely as net exporters of migratory pests. This possibility is being assessed for the biological control of Asian citrus psyllid in southern California and would be concomitant with bait-oriented ant control efforts in gardens that have demonstrated efficacy against pest ants [104].

Biotic interference can be assessed in the intended range of release by studying the effects of the resident biota on surrogate natural enemies that are ecological homologues of the classical biological control agent under consideration for importation. Such studies may predict in advance impediments to establishment, and experimental results may suggest possible countermeasures [99]. Another approach to identifying resistance factors that reduce natural enemy efficacy is to undertake meta-analyses of large data sets of pertinent descriptive or quantitative variables created from the published literature. From these data, “rules of thumb” can be developed and assessed for importance using statistical analyses and modeling to determine factors that may impede natural enemy establishment and impact [31,75].

### 3.4. Defensive Endosymbionts

Bacterial endosymbionts that confer resistance to natural enemies can have dramatic effects on attack rates with some strains of defensive bacteria conferring close to 100% protection from parasitoid attacks. These endosymbionts affect fitness (e.g., reproductive output, developmental times, insecticide susceptibility, and thermal tolerances [105]) of infested hosts and are passed between generations vertically from mother to offspring. The level of resistance is linked to different strains of the same species of endosymbiont, and protective effects are independent of host genotype. Genetic heterogeneity of protective endosymbionts could be due to their possession of bacteriophages [106] that produce protective toxins that kill larvae of endoparasitoids [105,107]. Additionally, removal of phages from defensive bacteria like *Hamiltonella defensa* (Enterobacteriaceae) has a direct adverse effect on their host, in this case the pea aphid, *Acyrthosiphon pisum* Harris (Hemiptera: Aphididae). Aphid fitness is depressed with time to reproduction being increased, reduced numbers of offspring, and production of smaller adults [107].

Parasitoids that are not adapted to countering specific strains of defensive endosymbionts can discriminate between infected and uninfected hosts. For aphid parasitoids, the quantity of aphid alarm pheromone differs between hosts with and without protective endosymbionts, and parasitoids show adaptive behavioral responses as a result. To successfully reproduce on defended hosts, parasitoids engage in superparasitism, the act of laying more than one egg in one host. Increased envenomation and levels of lipid-digesting teratocytes following multiple ovipositions into the host’s hemocoel may be responsible for overcoming microbial-based protection [108]. The obvious limitation to this strategy is that the reproductive potential of natural enemies is significantly diminished due to more eggs being laid inside fewer hosts.

A wide range of insects are protected from natural enemies via infection with inheritable protective endosymbionts [109], and very little is known about the responses of natural enemies to these defenses or what effects these endosymbiont-host-natural enemy interactions have on the efficacy of biological control programs, especially classical projects. Projects targeting pests with protective endosymbionts will need to locate natural enemies that can effectively counter the protective effects of endosymbionts. Past failures for some biological control programs may have resulted, in part, from an inability of imported natural enemies to overcome these endosymbiont-mediated defenses. Biological control programs targeting pests known to harbor protective endosymbionts (e.g., aphids and whiteflies) will need to identify bacterial species and strains responsible for host resistance prior to initiating foreign exploration efforts. Collection efforts will then need to target natural enemies that are co-adapted to specific endosymbiont strains whose hosts are being targeted for control to maximize the likelihood that efficacious candidate natural enemies are imported for potential future release.

### 3.5. Pest and Natural Enemy Coevolution

Pest and natural enemy coevolution are post-release processes that could affect long-term stability and efficacy of a classical biological control project [110]. Research focused on natural enemy biology and behavior has typically failed to investigate genetic variation in host use and responses by hosts to natural enemies [111]. Over time, post-release natural enemy population sizes, structure, and connectivity are affected by selection, genetic drift, and inbreeding, all of which may affect fitness, population growth rates, and potential host use patterns. Natural enemies may undergo rapid microevolutionary changes owing to selection, genetic drift, gene flow, mutation, and hybridization after release into new areas [111,112,113,114], but these processes are typically not studied, and effects are largely unknown. There is laboratory evidence to suggest that, at least for some *Trichogramma* spp. (Hymenoptera: Trichogrammatidae), hybridization between species can theoretically lower impact because incompatible matings result in fewer daughters. However, evidence for interspecific mating for these species in zones of sympatry is weak [115]. Local adaptations by released natural enemies and greater genetic variability at time of initial introduction may affect evolutionary trajectories, making the outcomes of post-release evolution unpredictable [47]. Alternatively, understanding genetic factors underlying certain natural enemy attributes, such as emergence times from diapause or onset of reproductive activity, may allow selection and exploitation of natural enemy biotypes with characteristics that maximize temporal overlap with target species thereby enhancing their impact because of improved synchronicity [116].

Better understanding of evolutionary processes has the potential to improve establishment rates and efficacy of natural enemies [114] and offset the potential for resistance development by pests. The “geographic mosaic theory of co-evolution” predicts that selective forces acting on interacting species vary depending on location, and populations in some areas may experience strong evolutionary interactions while in other places populations may be subjected to weak forces [117]. For example, localized host populations may have strong or weak resistance to attack by a natural enemy, resulting in variable pest control. This situation may be evolving in California walnut orchards where the highly successful suppression of the walnut aphid, *Chromaphis juglandicola* (Kaltenbach) (Hemiptera: Aphididae) by *Trioxys pallidus* Haliday (Hymenoptera: Aphidiidae) is now failing in some areas and pesticide use, hyperparasitism, and intraguild predation of aphid mummies cannot fully account for this decline in natural enemy efficacy [118].

The rate of evolution of resistance to natural enemy attack appears to be low. The one documented case involved increased encapsulation of eggs of the parasitoid *Mesoleius tenthredinus* Morley (Hymenoptera: Ichneumonidae) by the larch sawfly, *Pristiphora erichsonii* (Hartig) (Hymenoptera: Tenthredinidae), <30 years after it was introduced into Canada. The situation was remedied by introducing encapsulation resistant strains of *M. tenthredinus* from Bavaria [119]. The successful classical biological control program targeting Argentine stem weevil, *Listronotus bonariensis* (Kuschel) (Coleoptera: Curculionidae), a pasture pest in New Zealand with the parasitoid *Microctonus hyperodae* Loan (Hymenoptera: Braconidae) in 1991 appears to be failing as overwintering parasitism rates have declined by ~55% from 1994 to 2014 [120]. The reasons for this significant reduction in parasitoid efficacy are unknown but could be due to a thelytokous parasitoid attacking a sexually reproducing weevil and due to lack of genetic variation, the parasitoid cannot evolve to counter the host’s resistance response. Selection pressures of this type may be intensified because of the homogenous pastoral system in New Zealand and this simplified ecosystem benefits the pest because it is not subject to high levels of attack by other natural enemies and “background biological control” is not sufficient to ameliorate the pest’s resistance advantage [120].

## 4. Avoiding Avoidable Obstacles: Using New Tools in the Toolbox

Past analyses of unsuccessful biological control projects have focused on identifying potential causes such as establishment failure [31,80], lack of adequate control if natural enemies established [6,121], and biological, ecological, and behavioral traits that theoretically define a successful natural enemy [122]. Project failure analysis has pointed to the importance of climate matching [27], accurate taxonomy of pests and natural enemies [13], and high levels of host specificity, a trait considered important but, in the case of arthropod natural enemies, one that was often overlooked in favor of generalist natural enemies [122]. Uncertainty surrounding these key elements of a biocontrol program can be investigated, as there are sophisticated analytical tools to match climates (e.g., CLIMEX) that can be used to guide foreign exploration efforts for natural enemies. Powerful molecular tools are available to accurately identify species, an important element in finding host specific natural enemies. Similarly, advances in disciplines such as invasion biology and molecular genetics provide insight into factors affecting natural enemy establishment (e.g., propagule pressure), tools to identify the pest’s area of origin, and techniques on how to preserve genetic variability in natural enemy colonies started in quarantine (e.g., isolines). Perhaps the most significant recent advancement has been in understanding the interactive effects the microbiome of the target pest and the natural enemy have and how microbial level interactions affect host resistance and natural enemy efficacy (e.g., protective endosymbionts). Many of these tools need to be used simultaneously when developing a biological control program though perhaps the most underrated tool is simply perseverance during the establishment phase of initial natural enemy releases.

### 4.1. Applicability of New Technologies for Biological Control of Legacy Pests in California: Citricola Scale as a Case Study

Citricola scale, *Coccus pseudomagnoliarum* (Kuwana) (Hemiptera: Coccidae), is an exotic pest of citrus in California. Infestations on citrus were first detected in California around 1909, but it is likely that the scale invaded earlier than this [123]. The major economic damage caused to citrus by citricola scale is due to heavy infestations on branches that can cause die back and reduction in flowering and fruit set (*i.e.*, yield reductions), and honey dew and sooty mold contamination of picked fruit [124]. Citricola scale is an economic problem in California’s San Joaquin Valley (SJV) where >85% of citrus is grown on ~84,000 ha with average annual farm gate receipts of $1.6 billion (US) [125].

It was originally concluded that citricola scale, initially confused with brown soft scale, *Coccus hesperidum* L. (Hemiptera: Coccidae), was invasive, but its area of origin was unknown at time of first detection [123]. In addition to infesting citrus, its preferred host species in California, citricola scale was also recorded from pomegranates (*Punica granatum* L. [Lythraceae]), elms (*Ulmus* spp. [Ulmaceae]), walnuts (*Juglans* spp. [Juglandaceae]), buckthorn (*Rhamnus crocea* Nutt. [Rhamnaceae]), and hackberry (*Celtis occidentalis* L. [Cannabaceae]). Several striking biological differences eventually led citrus researchers to conclude that citricola scale was not a biotype of brown soft scale. First, in California, citricola scale is univoltine and brown soft scale is multivoltine. Second, the invasive Argentine ant, *L. humile* , does not readily tend citricola scale colonies to harvest honeydew, but actively tends brown soft scale for honeydew. Third, citricola scale produce offspring via eggs, while brown soft scale females give birth to live crawlers. Fourth, citricola preferentially infests older woody material, while brown soft scale is found more commonly on younger plant growth. Fifth, very few parasitoids are associated with citricola scale and biological control is typically poor in some areas, especially in the SJV. In comparison, the parasitoid fauna on *C. hesperidum* is much richer and often exerts substantial population control throughout the entire California range of this pest. Sixth, several misidentifications were made of citricola scale (*i.e.*, it was identified as *C. hesperidum*, *C. longulus*, and *C. elongatus* [123]). Misidentifications of a new invasive pest are not uncommon, especially if the species is undescribed, access to type specimens is difficult, and keys to described species are being used in an attempt to identify a newly established insect that is new to science. These observed biological differences, coupled with morphological differences, led to the description of a new species, *C. citricola* [126], which was separable from *C. hesperidum* mainly based on the number of antennal segments (eight in citricola scale and seven in brown soft scale [127]).

Quayle [123] presciently pointed out that this new species, *C. citricola*, may have been described previously from its native range, but that information was not available to California scientists, indicating that synonymization of citricola scale with an already described species was possible at some future point. This happened and *C. citricola* was synonymized with a described species from Japan, *Lecanium pseudomagnoliarum* Kuwana, collected from Oji Japan (near Tokyo) on trifoliate orange, *Poncirus trifoliata* (L.) (Rutaceae). Consequently, *C. citricola* was renamed *C. pseudomagnoliarum* (Kuwana) [128]. Curiously, Clausen [128] notes that morphological differences between *L. pseudomagnoliarum* and *C. citricola* exist but the significance of this was not elaborated upon. Clausen’s [128] fieldwork in Japan indicated that citricola scale was widespread in citrus growing areas but it was generally rare and consisted of low density non-damaging populations. Quayle [127] speculated that hackberry imported from Japan might have been the source of the invasive population in California.

Significant foreign exploration efforts were made in Japan for natural enemies of citricola scale that could be used in a classical biocontrol project targeting this pest in California [129]. Attempts to introduce and establish at least six species of citricola parasitoid over 1922, 1934, 1951, 1953, and 1985 failed, and in some instances, parasitoids could not be maintained in quarantine on colonies of California citricola scale [129]. Field surveys in Japan indicated that the parasitoid fauna associated with citricola scale could vary markedly depending on the species of host plant on which the scale was infesting [129].

Several hypotheses have been postulated as to why classical biological control of citricola scale in the SJV failed. These include: (1) citricola is immune to parasitoid attacks in its overwintering stage; (2) winters are too cold for citricola scale parasitoids and they die out; (3) univoltinism of citricola scale results in life stages susceptible to parasitism being absent for long periods of time and there are insufficient alternative hosts available for parasitoids to sustain themselves; and (4) hyperparasitism may reduce the population growth of parasitoids attacking citricola scale [130,131].

Kennett *et al.* [124] state that biological control of citricola scale in major citrus production regions of the SJV is negligible and urgently needed. The need for effective biological control of citricola scale in the SJV is increasing because broad-spectrum pesticides, organophosphates and carbamates, targeting other citrus pests (e.g., California red scale, *Aonidiella aurantii* Maskell [Hemiptera: Diaspididae]) have historically controlled citricola scale. These compounds are being replaced with new generation pesticides that may not be as effective as the compounds they are replacing [132]. Further, pesticide resistance has been detected in populations of citricola scale in the SJV weakening the efficacy of available products [133]. Increasing restrictions on the use of pesticides and resistance development has allowed citricola scale to re-emerge as a significant pest of citrus [134].

Kennett *et al.* [124] state that the following properties of the ideal hypothetical citricola parasitoid should be: (1) a parasitoid able to attack a range of citricola sizes in which parasitoid development is solitary in small (1.0 to 1.5 mm) hosts and gregarious in large hosts (1.75 to 4.0 mm). These attributes would promote parasitoid survival even though the scale is univoltine and only one size of life stage is present at any given time; (2) Parasitoids should be sourced from areas with a good climate match with the SJV, as hot summer temperatures could have a strong negative impact on the survivorship of species with poor climatic adaptations [124]; (3) Generalist scale parasitoids could be advantageous as they could use other pest scales as hosts should susceptible stages of citricola scale be unavailable at certain times of the year. This latter suggestion would need very careful scrutiny and consideration given the significant movement towards high host specificity for natural enemies of arthropod pests [5]. The Universal Chalcidoidea Database (http://www.nhm.ac.uk/research-curation/research/projects/chalcidoids/database/index.dsml) indicates that at least 45 different parasitoid species from five different families have been reared from citricola scale. Given this potentially rich species complex, the ideal citricola parasitoid according to Kennett *et al.* [124] may exist.

In comparison to the SJV, citricola scale is under good biological control in southern California and is not as problematic because parasitoids attacking black scale, *Saissetia oleae* (Olivier) (Hemiptera: Coccidae), which is a multivotine pest, spill off this host and exploit susceptible stages of citricola scale when they are present [124]. This does not happen in the SJV, as citricola scale immatures are too small when parasitoids attacking black scale are most active [135].

Biological control of citricola scale may now be emerging as an important control option again because of emerging issues with conventional control strategies that are reliant on pesticides. One step in this direction has been to use augmentative releases of black scale parasitoids for citricola scale control [131,134]. However, there are significant biological [136] and economic limitations to this approach because of the large numbers of parasitoids needed for release per acre. The use of banker plants, *Yucca* sp., infested with brown soft scales and parasitized by *Metaphycus* spp., the key parasitoid group attacking citricola scale in southern California [130], has been suggested as one way to increase parasitoid activity against citricola scale in citrus orchards in the SJV. Interestingly, this approach would require active encouragement of ants to tend brown soft scale colonies to prevent parasitoids from completely eliminating infestations on *Yucca* sp. plants (this idea is credited to R.F. Luck, L.D. Forster, and J.G. Morse, but it has not been field tested). Citricola scale is an excellent example of a legacy pest, and classical biological control of this important citrus pest in California should be revisited using modern tools. Suggested steps in developing a new biocontrol program targeting citricola scale with new tools are provided below.

### 4.2. Application of Molecular Tools to Determine Species Identities, Area of Origin, and Identity of Defensive Endosymbionts

The failure of establishment of citricola scale parasitoids from parts of the presumed native range may be indicative of the incorrect identification of the target pest in Japan. It is possible that past collection efforts were made from a scale species that was not the same species as that in California, a possibility hinted at by Clausen [128]. Accurate species identification for this project is imperative as all available published foreign exploration records indicate that natural enemy collections have only been made in Japan and any uncertainty over species identifications could be readily resolved via molecular work. Additionally, the native range of citricola scale is not well understood and needs to be better refined. The general area of origin is thought to include parts of China, Taiwan, Korea, and Japan, and citricola scale (*sensu latu*) in this vast area may be comprised of multiple species that are difficult to separate morphologically and are all considered to be *C*. *pseudomagnoliarum*. Molecular analyses of samples collected throughout the presumptive native range of citricola scale may help define the home range of this pest and could pinpoint the area within this large geographic region from which California’s population originated. Identification of the area from which California’s citricola scale population originated may assist with the location of natural enemies adapted to the invasive genotype present in California. The possibility of microbes protecting California populations of citricola scale from parasitoids should be investigated to determine if defensive endosymbionts are present and if they have the potential to affect the efficacy of different natural enemy species attacking citricola scale. If defensive endosymbionts are detected in citricola scale in California, these organisms may have contributed, in part, to past establishment failures of imported parasitoids from Japan. Collection efforts would therefore need to target natural enemies that are co-adapted to specific protective endosymbiont strains found in California citricola scale populations.

### 4.3. Climate Matching and Ecological Niche Modeling

Climate matching tools should be used to guide foreign exploration efforts for natural enemies of citricola scale in the native range, and ecological niche modeling could assess potential climate influences on the suitability of the SJV for natural enemy persistence when sourced from different areas within the presumed home range of citricola scale. The results of these modeling tools could be overlaid with molecular data used to determine the area of origin of California’s citricola scale population within the large native range, thereby further refining the geographic area, in which natural enemies are searched for, to one with both a good climate and genetic match.

### 4.4. Selecting Host Specific Natural Enemies, Preserving Natural Enemy Genetic Diversity in Quarantine, and Release Strategies to Increase Establishment Likelihood

The most host specific natural enemies of citricola scale need to be identified. This could be done either by studying the pest-natural enemy complex in their home range and importing potential best candidates into quarantine for host specificity testing, or by returning to quarantine candidate species for testing without *a priori* knowledge of their specificity. Polyphagous species should be excluded from consideration as biocontrol agents, and efforts should focus on species with high host specificity because they may have the greatest potential for establishing and controlling the target. In quarantine, the genetic diversity of collected natural enemies needs to be maintained and the use of isolines or isofamilies could be very important for preserving genetic variation. One way to do this would be to maintain separate natural enemy rearing cages with each cage representing a unique combination of collection location and time. This approach would preserve spatial and temporal genetic “snapshots” of biocontrol agents including those collected from the same locations but at different times. Genetic variation preserved in cages holding isolines or isofamilies would be reconstituted by removing individuals from these cages and introducing them into hybridization cages to facilitate panmictic mating. Resulting hybrid offspring with presumably increased levels of genetic diversity would be released. The number individuals released and frequency of releases will play an important role in establishment success. Multiple releases (>20) of moderate numbers (>800) of mated females per release that have been fed a carbohydrate (e.g., honey) or protein source (e.g., allowed to host feed) should be made at several locations (>10) where biotic resistance is low (e.g., ant control has been enforced) and resources supporting survival and reproduction are available. For example, releases should be made when host phenology is correct for natural enemy reproduction, hosts are abundant, and floral resources or some other food subsidy is available for newly released natural enemies and their anticipated offspring. Site security needs to be ensured to minimize preventable accidents such as pesticide sprays or pruning of trees which could accidentally eradicate incipient natural enemy populations.

## 5. Conclusions

Classical biological control of arthropod pests infesting perennial crops is an important pest management tool and often a key component of integrated pest management programs. However, there are some significant pests of tree crops grown in California that established prior to 1990 or have been present for more than 25 years that have not been successfully suppressed by natural enemies even though they were targets of biocontrol projects. We acknowledge that numerous reasons may exist for the failure of these programs. However, we contend that certain legacy pests may be good targets for classical biological control even if past efforts were unsuccessful. The development of new tools as discussed here could now provide opportunities to revisit old pest problems, and their application may increase the chances of successfully developing biocontrol programs. Citricola scale in California is an example of a legacy pest of citrus, which has been subjected to multiple biocontrol projects that have failed. Application of new tools, such as DNA-based analyses to determine species identities and areas of origin, climate matching, ecological niche modeling, and microbiome analyses, which were previously unavailable to scientists undertaking citricola scale biocontrol, have the potential to significantly influence the success of future projects targeting not only this pest but other legacy pest species as well.
